# Lessons Learned in Community Research Through The Native Proverbs 31 Health Project

**DOI:** 10.5888/pcd11.130256

**Published:** 2014-04-17

**Authors:** Caroline M. Kimes, Shannon L. Golden, Rhonda F. Maynor, John G. Spangler, Ronny A. Bell

**Affiliations:** Author Affiliations: Caroline M. Kimes, Shannon L. Golden, Rhonda F. Maynor, John G. Spangler, Wake Forest School of Medicine, Winston-Salem, North Carolina.

## Abstract

**Background:**

American Indian women have high rates of cardiovascular disease largely because of their high prevalence of hypertension, diabetes, and obesity. This population has high rates of cardiovascular disease-related behaviors, including physical inactivity, harmful tobacco use, and a diet that promotes heart disease. Culturally appropriate interventions are needed to establish health behavior change to reduce cardiovascular disease risk.

**Community Context:**

This study was conducted in Robeson County, North Carolina, the traditional homeland of the Lumbee Indian tribe. The study’s goal was to develop, deliver, and evaluate a community-based, culturally appropriate cardiovascular disease program for American Indian women and girls.

**Methods:**

Formative research, including focus groups, church assessments, and literature reviews, were conducted for intervention development. Weekly classes during a 4-month period in 4 Lumbee churches (64 women and 11 girls in 2 primary intervention churches; 82 women and 8 girls in 2 delayed intervention churches) were led by community lay health educators. Topics included nutrition, physical activity, and tobacco use cessation and were coupled with messages from the Proverbs 31 passage, which describes the virtuous, godly woman. Surveys collected at the beginning and end of the program measured programmatic effects and change in body mass index.

**Outcome:**

Churches were very receptive to the program. However, limitations included slow rise in attendance, scheduling conflicts for individuals and church calendars, and resistance to change in cultural traditions.

**Interpretation:**

Churches are resources in developing and implementing health promotion programs in Christian populations. Through church partnerships, interventions can be tailored to suit the needs of targeted groups.

## Background

American Indians bear an unequal burden of illness and death from cardiovascular disease compared with whites (1). Moreover, American Indians have high rates of cardiovascular disease risk factors, including hypertension, diabetes, obesity, physical inactivity, a diet that promotes heart disease, and tobacco use (2). Metabolic syndrome is also higher in this population as compared with its white counterpart (3). Although cardiovascular disease affects all US communities, American Indians die from this condition at younger ages than any other racial/ethnic group (4). In North Carolina, the 2004 through 2008 American Indian death rates for heart disease (207.7 per 100,000) and stroke (54.6 per 100,000) were both 10% higher and the diabetes death rate (138.0 per 100,000) was 70% higher than the rates for non-Hispanic whites (5). Disparities in cardiovascular disease risk factors for American Indians in North Carolina are similar to those seen nationally.

American Indian women have high rates of cardiovascular risk factors, including smoking, obesity, diabetes, physical inactivity, and unhealthy eating. The Strong Heart Study is a population-based longitudinal study that examined cardiovascular disease and its risk factors among American Indians (6). This study demonstrated that the prevalence of metabolic syndrome, a major risk factor for cardiovascular disease, was 56.7% for American Indian women compared with 43.6% for American Indian men enrolled in the study. Moreover, this 56.7% is much higher than the 23.1% prevalence of metabolic syndrome among women in the third National Health and Nutrition Examination Survey (1988–1994). Survey respondents were non-Hispanic whites, African Americans, and Mexican Americans) (7). Similarly, the Inter-Tribal Heart Project, a study of cardiovascular disease in American Indian women in Minnesota and Wisconsin, showed high rates of cardiovascular disease risk factors (8).

Community-based interventions, including the tribal programs participating in the WISEWOMAN (Well-integrated Screening and Evaluation for Women Across the Nation) initiative of the Centers for Disease Control and Prevention, are recognized as vital in addressing the significant cardiovascular disease burden in American Indian communities (9,10). These studies were conducted within various community settings, including schools, homes, and existing tribally operated community organizations (eg, community centers, health care facilities).

## Community Context

According to the US Census, North Carolina has the largest American Indian population of any state east of the Mississippi River. In 2012 estimates, American Indians comprise 1.9% of the state's population, or about 184,000 individuals (American Indian alone or in combination with other races). The Lumbee tribe, a state-recognized, nonreservation tribe with a population of about 55,000, is the largest tribe in the state and is mostly concentrated in Robeson County (11). This county is rural, has a high poverty rate, is racially and ethnically diverse, and has a large population of American Indians (39.0%) and African Americans (24.7%); 8.2% of all races report Hispanic ethnicity. Disparities are documented among the Lumbee in diabetes and cardiovascular disease-related health behaviors (12–14).

The Christian church and Christian faith are important components of Lumbee culture. The Baptist and Methodist faiths are the most prominent denominations in the county (15). Many studies show that the church is important in the delivery of cardiovascular disease-related health education (16–20). This type of intervention is understudied among American Indians. Thus, a church-based approach has the potential to reach a large number of underserved American Indians at high risk for cardiovascular disease.

## Methods

The Native Proverbs 31 Health Project was funded by the National Institute on Minority Health and Health Disparities as a feasibility study with the overall goal of developing, delivering, and conducting a preliminary evaluation of a community-based, culturally appropriate cardiovascular disease program for American Indian women and girls. The program was based on the Proverbs 31 “Virtuous Woman” chapter from the Bible and was conducted as a partnership between Native American Interfaith Ministries (Healing Lodge) and Wake Forest School of Medicine’s Maya Angelou Center for Health Equity and the Department of Family and Community Medicine. One of the co-investigators (R.A.B.) is an enrolled member of the Lumbee tribe and has led health-related research among the tribe for 20 years.

The study used a mixed-methods approach to achieve the following aims: 1) develop a community-based 4-month cardiovascular disease intervention for American Indian women and girls (aged ≥12 years) in North Carolina focusing on the themes adapted from Proverbs 31; 2) use a community health and lay health educator model to implement the intervention in 4 Lumbee Indian churches; 3) evaluate the intervention using measures including change in diet, physical activity, and harmful tobacco use. The study was approved by the Wake Forest Health Sciences Institutional Review Board.

### Phase 1, formative research process

In the initial phase of the project, we conducted formative research designed to inform the development of the intervention and gain a better understanding of the population in which the intervention was to be developed. The formative process began with a review of successful church-based programs and evidence-based guidelines in the development of the intervention. We then met with key church leaders from the community to describe study goals and assess which churches would best serve as sites for the program. Church leaders suggested several churches that they thought would be well-suited to our proposed program.

Next, we conducted an assessment of these churches and their infrastructures pertaining to health issues. The assessment included the following: 1) availability of exercise equipment and facilities, including a designated on-site walking trail; 2) existence of policies focused on health issues, such as on-grounds smoking bans and provisions for healthy foods at church events, 3) availability of staff dedicated to health issues (eg, parish nurse, health educator); 4) past participation in health programs or activities; and 5) existence of family-based programs and events. After a review of these factors, we selected churches to participate that had similar histories, congregation sizes sufficient to provide an adequate sample of American Indian women, and geographic distance from each other. We then conducted focus groups (1 per church, 5 to 9 participants per focus group) with members and key leaders of the selected churches. The goals of the focus groups were to 1) assess the level of understanding of cardiovascular disease risk factors, 2) determine the level of receptivity of a church-based cardiovascular disease prevention program, 3) identify the key elements of Lumbee culture that are important for the development of health messages, 4) test messages adapted from the Proverbs 31 verses to determine the level of receptivity of the church members to the relationship between the verses and health behaviors, and 5) determine the appropriateness of the design elements and delivery style (eg, artwork, words and phrases, photography). We were also sensitive in the development of the intervention messages pertaining to harmful tobacco use, given the strong cultural and economic ties that the Lumbee have with tobacco. Focus groups were audio recorded but not transcribed. The research team took detailed notes and reviewed the recordings to assess how to modify the intervention for the churches. Modifications included decreasing the length of the intervention from 6 to 4 months, reducing the frequency of classes to 1 time per week, adding more class topics on exercise and nutrition, and changing the study sample. Originally we hoped to design the intervention for mothers and their daughters, but focus groups felt that American Indian adolescents and women aged 12 and older would be best.

### Phase 2, intervention

In the second phase of the study, the research team and community partners developed the intervention for Lumbee women that was tailored for Lumbee culture and demonstrated how the verses in Proverbs 31 translate into healthy behaviors (Table 1). Data were collected at 2 primary intervention sites and 2 delayed intervention sites. The churches were selected to participate in the primary and delayed intervention based on their geographic distance from each other.

**Table 1:** Biblical Passages From Proverbs 31 and Potentially Relevant Health Implications

Proverbs 31 Verse	Potential Health Implications
12: She will do him (husband) good and no harm	Is concerned about the health of her family.
13: She . . . works willingly with her hands	Prepares healthful foods, gardening
14: She . . . brings food from afar	Prepares healthful foods from grocery stores (fruits, vegetables)
15: She . . . gives meat to her household, and a portion to her maidens	Prepares healthful meats, works with daughters to learn healthful practices
16: . . . with the fruit of her hands, she plants a vineyard	Prepares a garden for fresh fruits and vegetables
17: She girds her loins with strength and strengthens her arms	Participates in physical activity and exercise
20: She stretches out her hand to the poor, she reaches forth her hand to the needy	Participates in community health efforts, especially for those in need
26: She opens her mouth with wisdom	Teaches her family healthful practices (eating healthy, exercising, avoiding tobacco)

The 4 groups were administered questionnaires at baseline and 4 months. After the 4-month assessment of the primary intervention sites, the same program was offered at the delayed intervention sites. Inclusion criteria for participation in the intervention included being a member of the Lumbee tribe or a Lumbee family, female aged 12 or older, and able to engage in low-impact physical activity.

The intervention involved a 4-month, weekly health education program taught by trained lay health educators from each church. Lay health educators were identified in the churches through word-of-mouth and through referral from church leadership. They completed an application that was reviewed by the study team. The lay health educators completed the Collaborative Institutional Training Initiative modules for human subject research, as required by the Wake Forest Health Sciences Institutional Review Board, and were trained by the research team and community partners to deliver the intervention. Training involved a one-half day session in the study county where the study objectives were discussed, and lesson plans developed by the study team were reviewed. Teams of lay health educators and field staff then recruited participants for the program from their congregations and communities by using flyers and attending local events like women’s programs and blood drives. Then lay health educators, in conjunction with church leaders, decided on the program schedule. Each session of the program lasted approximately 2 hours and was held in the evening. Each participant was presented with a notebook at the beginning of the program and provided with subject matter weekly.

The curriculum included presentations, group discussions, handouts on 11 topics (Box), and several review sessions. Resources used to develop the health education messages were drawn from trusted organizations such as the American Heart Association; the National Heart, Lung, and Blood Institute; and the North Carolina Department of Public Health. The team also relied on their personal research experience in the community and from the insight of the community partners. The intervention was modeled on the Heart disease and stroke Education Awareness Rapid response Treatment adherence Quality Enhancement through Science Translation (HEARTQUEST) curriculum, developed as part of a project funded by the National Heart, Lung, and Blood Institute that was part of the Enhanced Dissemination and Utilization Centers (R.A. Bell, principal investigator).

**Box. Intervention Session Topics from The Native Proverbs 31 Health Project**

Heart Disease and Stroke

High Blood Pressure, Salt and Sodium

Eat Less Fat, Saturated Fat, Trans Fat, and Cholesterol

Grocery Store Tour

Diabetes and Obesity

Getting More Fruits and Vegetables in Each Day

Making Heart-Healthy Eating a Family Affair

Eat in a Heart-Healthy Way, Even When Money is Tight

Being Active Throughout the Day

Avoiding Harmful Tobacco Exposure

Advocacy: Making Heart-Healthy Changes in Your Community

Review Sessions

Each session had specific elements to be covered by the lay health educators using lesson plans. The elements included a review of the Proverbs 31, a “Proverbs 31 Pledge,” a physical activity or stretching time, a dietary lesson, and a class activity or worksheet. Some flexibility was given to the lay health educators in the delivery of these elements. For example, 1 church had a “walking club” session before the class.

Participants were asked to make weekly “Proverb 31 Pledges” where they would commit to make 1 change in their lifestyles based on the lesson from each week. Pledges were reviewed at the next session and women discussed successes and pitfalls regarding their pledges during the previous week. Grocery store tours were arranged with a regional grocery store chain in collaboration with a registered dietitian. We provided tools and materials to participants as incentives throughout the program, including notepads, pens, pedometers, calculators, cookbooks, and tote bags all bearing the study logo. In addition, church-wide efforts were made to support and promote the program. Church bulletin inserts related to the curriculum were distributed congregation-wide, as were fans printed with information about healthy living.

Lay health educators collected weekly attendance and monthly evaluations from participants. Additionally, lay health educators evaluated the success of each session weekly. These processes allowed the research team to monitor the activities of each church and provide necessary resources in a timely manner.

### Intervention data collection and study measures

After obtaining informed consent and assent from participants, the study team collected questionnaire data at the churches before the first scheduled session and again at the conclusion of the program. Questionnaires took 30 to 45 minutes to complete. Measures included 1) demographics, including sex, ethnicity, age, marital status, household size, formal education, and annual household income (from adults only); 2) perceived health status; 3) dietary intake including self-efficacy and readiness to adopt healthful eating behaviors; 4) physical activity, including self-efficacy and readiness to adopt healthy physical activity behaviors; 5) current and tobacco use; 6) depressive symptoms; 7) self-esteem; 8) religiosity. After questionnaire completion, height and weight were measured and body mass index computed. The primary outcomes were changes in dietary intake, physical activity, and tobacco use. Secondary outcomes included changes in body mass index, self-efficacy, and self-esteem (Tables 2 and 3).

**Table 2:** Baseline Demographic and Health Characteristics of Adult American Indian Women Participants by Intervention Group, The Native Proverbs 31 Health Project, 2011–2012^a^

Characteristic	Primary Intervention Group (N = 64), N (%)	Delayed Intervention Group (N = 82), N (%)	Total (N = 146), N (%)
**Demographic**			
American Indian, non-Hispanic	62 (97)	77 (94)	139 (95)
Age, y, mean (SD)	49.1 (14)	49.9 (15)	49.6 (14)
Married	37 (58)	54 (66)	91 (62)
**Household size**			
1	17 (27)	13 (16)	30 (21)
2–4	40 (63)	59 (72)	99 (68)
≥5	7 (11)	10 (12)	17 (12)
**Formal education**			
Less than high school graduate	2 (3)	1 (1)	3 (2)
High school graduate	24 (38)	22 (27)	46 (32)
Some college	38 (59)	59 (72)	97 (66)
**Annual household income, $**			
<25,000	30 (47)	22 (27)	52 (39)
25,000–49,999	16 (25)	24 (29)	40 (30)
50,000–74,999	5 (8)	14 (17)	19 (14)
≥75,000	7 (11)	15 (18)	22 (17)
**Attends religious services at least once per week**	55 (86)	53 (65)	108 (75)
**Health**			
**Self-rated health**			
Excellent, very good, or good	48 (75)	76 (93)	124 (85)
Fair or poor	16 (25)	6 (7)	22 (15)
**Tobacco**			
Current user	7 (11)	13 (16)	20 (14)
Former user	16 (25)	13 (16)	29 (20)
**Body mass index, kg/m^2^**			
Normal weight, 18.5–24.9	1 (2)	12 (15)	13 (9)
Overweight, 25.0–29.9	20 (31)	22 (27)	42 (29)
Obese, ≥30	43 (67)	48 (59)	91 (62)
**Prior diagnosis**			
Type 2 diabetes	10 (16)	8 (10)	18 (12)
High blood pressure	26 (41)	28 (34)	54 (37)
High cholesterol	24 (38)	31 (38)	55 (38)

a Values are expressed as number (%) unless otherwise indicated.

**Table 3:** Baseline Demographic and Health Characteristics of Youth Female American Indian Participants by Intervention Group, The Native Proverbs 31 Health Project, 2011–2012^a^

Characteristic	Primary Intervention Group (N = 11), N (%)	Delayed Intervention Group (N = 8), N (%)	Total (N = 19), N (%)
**Demographic**			
American Indian, non-Hispanic	11 (100)	8 (100)	19 (100.0)
Age, y, mean (SD)	14.5 (1.7)	15.1 (1.8)	14.7 (1.7)
**Formal education**			
8th grade or less	5 (45)	3 (38)	8 (42)
9th to 12th grade	6 (55)	5 (62)	11 (58)
**Attends religious services at least once per week**	11 (100)	4 (50)	15 (79)
**Health**			
**Self-rated health**			
Excellent, very good, or good	9 (82)	8 (100)	17 (89)
Fair or poor	2 (18)	0	2 (11)
**Tobacco**			
Current user	0	0	0
Former user	1 (10)	1 (13)	2 (11)
**Body mass index, kg/m^2^**			
Normal weight, 18.5–24.9	2 (18)	6 (75)	8 (42)
Overweight, 25.0–29.9	6 (55)	1 (13)	7 (37)
Obese, ≥30	3 (27)	1 (13)	4 (21)
**Prior diagnosis**			
Type 2 diabetes	0	0	0
High blood pressure	0	0	0
High cholesterol	0	0	0

a Values are expressed as number (%) unless otherwise indicated.

## Outcome

Conducting community-based research or developing community-based programs is an ongoing process, involving constant communication between all partners and flexibility to accommodate developing needs in the community. Over time, the process has had both successes and opportunities for improvement.

Communication was critical for successful implementation of the elements of our study. Our primary study team is more than 100 miles from the study county where our community partners were. The study team hired an on-site Lumbee field coordinator whose primary role was to link the study team with partners at all times and keep communication flowing to advance the program. She kept the study team informed on local news and events and attended multiple community functions to represent the study team and network with partners. The staff of the Healing Lodge, trained specialists in health education and community health, were a valuable asset for the study by connecting the study team with key church leaders in the community. They reviewed the curriculum and provided critical feedback on how to format materials for the American Indian population. Additionally, they were present at each lay health educator class and helped to facilitate classes as needed.

There were several successes in community engagement. One main success was that the churches readily welcomed the pairing of health messages with the Proverbs 31 verses. Because classes were led by parishioners in the church, the community felt a sense of ownership of the program, even though attendance was slow to increase. One church formed a walking group that has continued postintervention. The churches that participated in the study were all rural, with limited availability of structured exercise facilities and safe walking areas, so the church became a safe venue to support physical activity. Another church drafted a nonsmoking policy for church grounds. The healthy living idea spread through many of the churches and entire families became involved.

Through the process, we faced challenges in initiating and sustaining enthusiasm to complete participation in all sessions. Attendance among participants surveyed at baseline averaged 27% between both primary intervention churches. Interest in the intervention increased slowly over time as participants invited others to join them. However, when collecting baseline data, we could not extend enrollment indefinitely and attendance was tracked for surveyed participants only. Also, for churches, adding additional meetings or activities to busy schedules can bring unexpected delays in curriculum delivery. After session cancellations from holiday events, funerals, and in the case of 1 church, 4 consecutive weeks of revival services, momentum was slowed. Many participants had demands on their time requiring high fast-food and processed-food consumption, or conversely, pressure from their families to cook extensive meals of “traditional food” often including high-fat products like whole milk, lard, cured pork, and deep-fat–fried foods. Adopting low-fat alternatives was included in the curriculum, but families of participants were often resistant to this change, and access to or affordability of high-quality products was not always possible in this rural setting. Effective change will take more time than a 4-month intervention can offer.

An additional challenge was that although the churches were geographically isolated in a large county (921 square miles), it is unclear how much interaction occurred among study participants from different churches. This is not something we were able to measure, and we tried to select churches that were distributed throughout the county.

We experienced shifting social and political climates at the time of our intervention. During implementation, there were changes in tribal leadership, which made it difficult for the study team to keep the tribe informed of and engaged in our activities. The study team was persistent in establishing relationships with the tribal government and maintaining a steady flow of communication about the project’s activities. We also experienced change in leadership at one of the intervention sites when a prominent member confessed to illegal behavior and left the church.

## Interpretation

Several community-based issues presented difficulties in implementing this intervention, including slow start-up, scheduling problems, and resistance to stray too far from traditional food and preparation methods. These issues made it difficult for some women to participate and implement lifestyle changes. However, even with these struggles, we were able to meet our objectives and engage 4 churches to implement the program. We were able to successfully determine feasibility, and this model could be adapted for use by churches in other communities. For this program to be sustained long-term elsewhere, it will be vital to have community buy-in, input, and leadership. This program could be adapted for communities with limited funds. Although it is difficult to calculate an exact dollar amount, the cost of the program is low, given that the curriculum has been developed and lay health educators would volunteer their time. The curriculum could easily be expanded to other Bible passages, and there are many biblically-based books that focus on healthy living that could be resources.

The development of our intervention focused on a biblical theme was aided by the strong Christian influence in the Lumbee community. Successful implementation would be more challenging in communities less receptive to such a focus, particularly in Native communities that have traumatizing histories with the Christian church and Christian missionaries.

Churches provide an opportune setting for intervention delivery because many in the congregation share common values and interests and the structure of classes is well-received. Curriculum can easily be promoted with media such as church fans and bulletin inserts because their use is common practice already. However, when designing and implementing community-based programs, it is not possible to anticipate changes in the social or political climate that might have an effect on the intervention. Community-based programs should be flexible in their design so that adjustments can be made as unforeseen circumstances arise. Even with these limitations, community partners, like churches, are valuable resources when developing and executing tailored health education and disease prevention programs in Christian populations.
